# Corona VRus Coaster: the virtual reality roller coaster of the proteins of SARS-CoV-2

**DOI:** 10.1007/s00894-025-06455-z

**Published:** 2025-08-06

**Authors:** Alejandro Seco-González, César Porto, Ángel Piñeiro, Rebeca Garcia-Fandino

**Affiliations:** 1 Centro Singular de Investigación en Química Biolóxica e Materiais Moleculares (CIQUS), Departamento de Química Orgánica, Universidade de Santiago de Compostela, Santiago, Spain; 2 Departamento de Física Aplicada, Facultade de Física, Universidade de Santiago de Compostela, Santiago, Spain

**Keywords:** Virtual reality, SARS-CoV-2, Protein visualization, Molecular structures, Immersive technology

## Abstract

**Context:**

The COVID-19 pandemic, caused by SARS-CoV-2, has driven significant research into the virus’ structural components to elucidate its infection mechanisms and advance therapeutic strategies. However, the intricate architecture of viral proteins presents challenges for researchers and students in structural biology. To address this, we developed Corona VRus Coaster, a virtual reality (VR) application designed to facilitate molecular visualization. This tool enables immersive exploration of over 2000 viral proteins, including key structures such as the spike protein, nucleocapsid protein, main protease, and RNA-dependent RNA polymerase. Users can “ride” along the molecular backbones of these proteins, offering a distinctive way to explore their three-dimensional organization. Hand-tracking technology provides an intuitive interface for real-time interaction, while multiple visualization and navigation modes enhance the exploration experience. This application offers a novel and engaging approach to structural biology, supporting both research and education by making molecular structures more accessible and interactive. Corona VRus Coaster is available for download at http://mduse.com/coronavruscoaster.

**Methods:**

Corona VRus Coaster was developed using Unity3D and integrates molecular visualization frameworks such as the Molecular Rift toolkit for VR rendering. Protein structures were sourced from the Protein Data Bank (PDB) and converted into VR-compatible formats using Open Babel and PyMOL. The application employs hand-tracking technology via the Meta Interaction SDK to facilitate direct manipulation of molecular structures. The visualization modes incorporate different molecular representations, including ribbon, surface, and space-filling models, rendered using GPU-accelerated shaders. Navigation is enabled through VR motion controllers and gaze-based selection mechanisms. The modular software architecture allows seamless integration of newly resolved protein structures, ensuring flexibility for future molecular visualization applications.

**Supplementary Information:**

The online version contains supplementary material available at 10.1007/s00894-025-06455-z.

## Introduction

The COVID-19 pandemic, caused by the SARS-CoV-2 virus, has significantly impacted global health, economies, and daily life [1]. Since the onset of the pandemic, an extensive amount of research has been focused on understanding the virus’ structure and function, particularly its protein components [2, 3]. In response to the urgent need for detailed structural information on SARS-CoV-2 proteins, the scientific community launched a coordinated effort to rapidly generate and share data. International collaborations were formed, pooling resources and expertise to accelerate the pace of research. Databases such as the Protein Data Bank (PDB) played a crucial role in this process [4]. The PDB, a repository for the three-dimensional structural data of large biological molecules, became an essential resource for researchers worldwide. As new structures of SARS-CoV-2 proteins were resolved, they were promptly deposited into the PDB, making them freely accessible to the global scientific community [5]. To further support this effort, additional platforms such as PDBj [6] or COV3D [7] have also been developed. Researchers utilized a variety of techniques to study these proteins, resulting in a diverse array of structural information. High-resolution structures of the spike protein, the main protease, RNA-dependent RNA polymerase, and other viral components were rapidly determined and shared. This accessibility has facilitated rapid advancements in understanding and combating COVID-19, playing a crucial role in the development of vaccines, antiviral drugs, and diagnostic tools [8]. However, despite the availability of these structures, their complexity often presents significant challenges in terms of visualization and comprehension, particularly for students, educators, and even researchers.

Virtual reality (VR) has emerged as a powerful tool in overcoming these challenges by providing immersive, interactive experiences that allow users to engage with three-dimensional representations of molecular structures in ways that traditional methods cannot. Originally developed for gaming and entertainment, VR has expanded into areas such as education, healthcare, and scientific research, offering new ways to interact with digital content [9]. During the COVID-19 pandemic, VR has proven invaluable in various contexts, from enhancing healthcare delivery and mental health support to facilitating remote collaboration [10–13]. One of its most promising applications, however, lies in the exploration of SARS-CoV-2 protein structures [14]. Traditional two-dimensional representations and static models often fall short in conveying the full complexity of molecular structures, making it difficult to grasp the spatial arrangements and interactions that are essential for understanding their biological functions. VR addresses this limitation by immersing users in a virtual environment where they can manipulate detailed 3D models of proteins. This interactive approach not only aids researchers in identifying potential therapeutic targets but also enhances the educational experience. By enabling students and scientists to explore the intricate details and spatial relationships of these proteins, VR provides a level of understanding of molecular mechanisms that is challenging to achieve through conventional visualization methods [15]. This hands-on, intuitive interaction with molecular data can lead to more effective comprehension and retention of complex concepts, making VR a valuable tool in biochemistry, molecular biology, and scientific education.

Recent advances in VR hardware have further broadened its applications, extending beyond entertainment into critical areas of scientific research, particularly in structural biology and drug discovery. Devices such as the Meta Quest and HTC Vive provide highly immersive experiences through high-resolution displays and precise motion tracking, enabling users to engage directly with complex 3D molecular models [16]. Meta Quest headsets, with their standalone functionality and accessibility, have emerged as a popular choice for scientific applications due to its ease of use and affordability. However, despite the rapid progress in hardware, the development of software specifically designed for molecular visualization in VR has been more gradual. Several existing tools, such as Nanome [17], Molecular Rift [18], VMD [19], and UnityMol [20], have demonstrated the promise of VR for exploring biomolecular structures, enabling users to manipulate and analyze 3D models of proteins and other biomolecules. Additionally, more accessible applications, such as ProteinVR [21] and VRmol [22], have broadened the reach of VR by utilizing cardboard-based platforms, which allow users to visualize molecular structures using standard smartphones. While these tools have been valuable in making VR-based molecular visualization more accessible, they often come with significant trade-offs. The limited processing power and graphical capabilities of mobile devices result in a less immersive experience, which can compromise the exploration of highly detailed molecular structures. Moreover, a poor experience with such low-cost solutions could lead to undervalue the true potential of VR in scientific research, possibly discouraging further adoption of more advanced systems.

Thus, despite the existence of these applications, the overall number of specialized VR tools remains relatively small. This is particularly evident in the context of the COVID-19 pandemic, where the rapid influx of structural data on SARS-CoV-2 proteins has highlighted the need for more sophisticated tools. As the demand for interactive, high-resolution molecular visualization grows, there is a clear opportunity to develop new applications that offer deeper, more engaging experiences. Such tools would not only enhance the scientific understanding of complex molecular structures but also help bridge the gap between accessibility and the high-level precision required for advanced research. Recognizing this need, we introduce Corona VRus Coaster, a novel VR application designed specifically for the visualization of SARS-CoV-2 protein structures. Unlike traditional static visualization tools, Corona VRus Coaster allows users to “ride” along the molecular backbones of viral proteins, simulating a roller coaster experience through the intricate details of these macromolecules. This unique approach provides a fresh and dynamic way to explore the protein topology, helping users to gain a deeper understanding of the spatial relationships and structural features that are essential for their biological function. By offering an immersive, engaging experience, Corona VRus Coaster fills an important niche in VR-based molecular visualization, making it a valuable tool for both research and education in the study of SARS-CoV-2.

## Materials and methods

Corona VRus Coaster was developed using the Unity3D engine, a robust platform widely employed for creating 3D and VR applications. The primary scripting was done in C#, the native programming language of Unity, allowing for efficient real-time interaction and VR integration. For molecular visualization, we utilized UnityMol [23], which was incorporated to handle the PDB file processing. UnityMol is responsible for reading the PDB files, determining atomic bonds, and generating the cartoon representations of proteins. This approach enables accurate molecular visualization within the VR environment.

The application was designed to run on the Meta Quest 2 headset, leveraging its hand-tracking capabilities. The hand-tracking system, enabled through the Meta Interaction SDK, allows users to interact with the environment without controllers by detecting hand gestures through the built-in cameras. This feature is critical to providing a more immersive and intuitive experience.

**Fig. 1:**
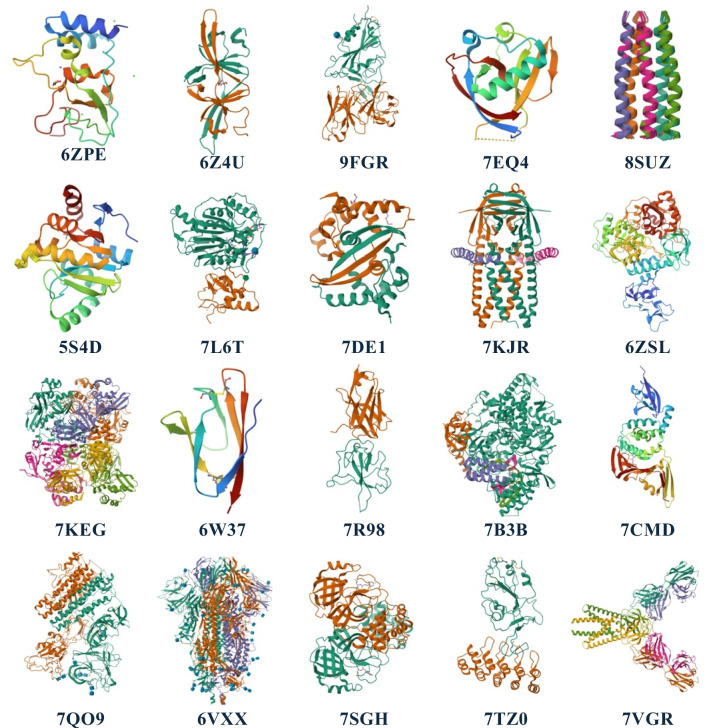
Preloaded SARS-CoV-2 protein structures available in the Corona VRus Coaster application. The 20 structures selected represent a range of viral components important for infection and replication, including multiple spike protein conformations (PDB IDs: 6VXX, 9FGR, 7TZ0), nonstructural proteins such as Nsp10 (PDB ID: 6ZPE), Nsp16/10 (PDB ID: 7L6T), and Nsp3 macrodomain (PDB ID: 5S4D), as well as the RNA-dependent RNA polymerase (PDB ID: 7B3B), the nucleocapsid protein (PDB IDs: 7DE1, 7R98), the main protease (PDB ID: 7CMD), and accessory proteins such as Orf9b (PDB ID: 6Z4U), Orf3a (PDB ID: 7KJR), and M protein (PDB ID: 7VGR). Other included structures are the envelope protein (PDB ID: 8SUZ), helicase (PDB ID: ZSL), and NendoU NSP15 (PDB ID: 7KEG). Additionally, the application provides access to over 2000 more structures available for real-time exploration

For accessing and displaying protein structures, the application integrates with the PDB databank [4] to retrieve structures in PDB format. These structures are dynamically loaded through a JSON file, which contains the PDB codes and metadata for over 2000 protein structures related to SARS-CoV-2. The JSON file acts as a central index, managing the initial set of 20 preloaded proteins (Fig. 1, with PDB ID codes: 6ZPE, 6Z4U, 9FGR, 7EQ4, 8SUZ, 5S4D, 7L6T, 7DE1, 7KJR, 6ZSL, 7KEG, 6W37, 7R98, 7B3B, 7CMD, 7QO9, 6VXX, 7TZ0, 7VGR) and enabling users to download and visualize more than 2000 additional structures in real time. The JSON structure is designed to be easily expandable for future updates, allowing new proteins and datasets to be added to the application without significant modifications to the core system. This modular approach provides flexibility for ongoing enhancements, ensuring that the application can evolve alongside emerging research and additional structural data. Additionally, a user interface was developed using Unity’s UI Toolkit to facilitate the selection and exploration of proteins. The UI includes a carousel-style menu, where users can browse proteins in groups of 20, navigate between different “pages” of proteins, and dynamically download additional structures. These interactions are seamlessly integrated with the hand-tracking system, enhancing user experience by allowing intuitive gestures for navigation and selection.

## Results and discussion

**Fig. 2:**
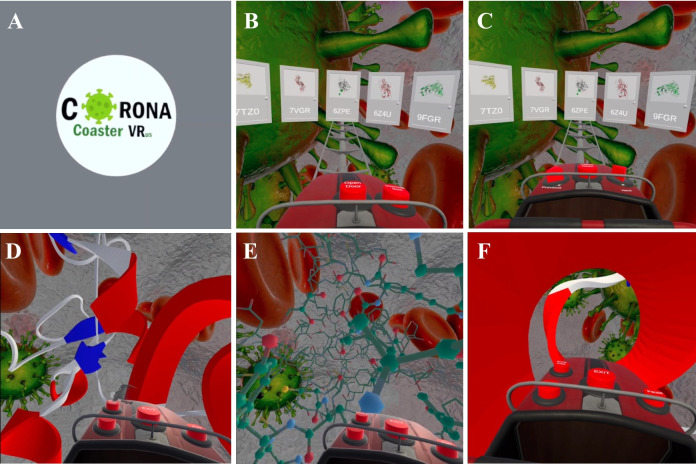
Screenshots showcasing various features of the Corona VRus Coaster application for visualizing SARS-CoV-2 molecular structures. **A** Application name and logo. **B** Initial 3D carousel menu for protein structure selection. **C** Arrows allow navigation between structures in the carousel. **D**–**E** Roller Coaster Navigation through a protein backbone visualized in Cartoon style (**D**) and Ball and Stick style (**E**). **F** Fly mode enabled during navigation, allowing free movement through the molecular structure

The Corona VRus Coaster, a VR application developed for the visualization and interactive exploration of SARS-CoV-2 protein structures, is introduced here. This application allows users to navigate through molecular backbones in a VR environment, providing a novel perspective on the spatial organization of viral proteins (Fig. 2, Supplementary Video). Designed to be compatible with standard VR headsets, the application leverages hand-tracking technology to enhance user interaction with the protein structures without the need for external controllers.

### Selection and visualization of protein structures

The current version of Corona VRus Coaster includes access to over 2000 protein structures associated with SARS-CoV-2. For ease of use, the first 20 structures are preloaded into the application and available for offline use after the initial download (Fig. 1). These proteins were selected to represent a balance between structural diversity and relevance, focusing on viral components crucial for infection and replication. Some of the key structures included in this set are multiple conformations of the spike protein (PDB IDs: 6VXX, 9FGR, 7TZ0), nonstructural protein 10 (PDB ID: 6ZPE), the RNA-dependent RNA polymerase (PDB ID: 7B3B), the main protease (PDB ID: 7CMD), and the nucleocapsid protein (PDB IDs: 7DE1, 7R98). The goal of this selection is to cover essential viral mechanisms, offering users a comprehensive view of key molecular targets while ensuring diversity in the structural features available for examination.

Inside the application, protein structures are displayed in a 3D carousel menu with visual snapshots of the available proteins (Fig. 2B–C). Users can select proteins using hand-tracking technology, performing a “pinch” gesture (joining thumb and index finger) to pick the desired structure. This interaction is made possible through the Meta Quest 2’s built-in cameras, allowing seamless navigation through the protein library.

### Access to additional protein structures

Beyond the preloaded set of proteins, Corona VRus Coaster offers dynamic access to over 2000 additional SARS-CoV-2 protein structures. These structures are not integrated in the application, but can be accessed in real-time using the “Download more” button located in the virtual kart (Fig. 2B–C). Upon selection, the application retrieves the necessary data from external databases, processes the 3D molecular structures, and renders them in the virtual environment. This dynamic functionality is managed via a JSON file that stores the PDB codes and metadata for all available proteins, enabling efficient updates and seamless access to new datasets.

Proteins are presented in groups of 20 within a carousel, and users can navigate between pages using arrow buttons located on the virtual kart (Fig. 2C). This browsing system allows for effective navigation through the large dataset without overwhelming the user with excessive initial data. Please note that while the 20 preloaded protein structures are available offline after installation, an active internet connection is required at runtime to retrieve additional structures from the Protein Data Bank.

### Interactive exploration and visualization modes

Once a protein is selected, users can engage with it using several interactive modes. The player is seated in a virtual kart equipped with three buttons: two arrows to switch between pages of protein structures, and an “Open Door” button to enter the navigation mode (Fig. 2C). After selecting a protein, the kart and tracks rotate to position the user in front of the chosen structure. By pressing the central button, the navigation mode begins, allowing the user to travel through the backbone of the protein, simulating a roller-coaster-like experience (Fig. 2D).

In navigation mode, several additional buttons are available, including an Exit button to return to the main menu, a Pause button to stop the navigation and enable the user to look around by moving their head in 360°, a “Fly” button that allows the kart to be removed from the tracks to freely explore the protein (Fig. 2F), and a Cartoon/Ball and Stick button to toggle between two visualization styles. The Cartoon mode displays simplified representations such as alpha-helices and beta-sheets, while the Ball and Stick mode provides a more atomistic view, allowing for detailed examination of the protein’s atomic structure (Fig. 2F).

As virtual reality roller-coaster experiences inherently involve dynamic movement through 3D environments, cybersickness remains a concern for some users, primarily due to sensory conflicts between visual and vestibular inputs [24, 25]. To address this, the current version of Corona VRus Coaster includes several implemented features designed to minimize cybersickness risk: default moderate navigation speeds, smoothed acceleration profiles, the ability to pause navigation at any time, and the option to switch into a free-flying exploration mode. These features aim to reduce abrupt visual motion and allow users to control their experience according to personal tolerance. In addition, recent research has explored hardware-based solutions such as bone-conduction vestibular stimulation to mitigate cybersickness symptoms [26], which may represent an interesting avenue for future development of the application.”

### Implications and future developments

The Corona VRus Coaster application presents a novel and dynamic approach to the visualization of SARS-CoV-2 proteins, offering new possibilities for exploring the complex molecular architecture of the virus. By integrating immersive VR with interactive molecular navigation, the application provides an alternative to traditional methods of protein visualization, particularly in educational and research environments. The ability to intuitively explore 3D protein structures in real-time enhances comprehension and provides users with a more engaging and insightful experience.

As research on SARS-CoV-2 continues, future updates to the application will aim to include newly identified protein structures, further expanding the library of available molecules. Additionally, refinements in user interaction and functionality, guided by user feedback, will be implemented to improve both the educational and research applications of the tool. The modular nature of the application, supported by a flexible JSON-based data management system, ensures that new datasets can be easily integrated, allowing the tool to evolve alongside the growing body of scientific knowledge.

Looking ahead, Corona VRus Coaster represents a significant step towards the broader adoption of immersive technologies for molecular visualization. The potential applications extend beyond SARS-CoV-2, offering a platform that could be adapted to other areas of molecular biology and drug discovery. By enabling a deeper understanding of protein structures, this tool may contribute to the development of new therapeutic strategies, not only for COVID-19 but for protein-targeted diseases as well. This application is available at http://mduse.com/coronavruscoaster/. By continuing to innovate and refine these immersive visualization tools, we aim to contribute to the development of future technologies that will support scientific discovery and education in molecular biology.

## Supplementary Information

Below is the link to the electronic supplementary material.

Supplementary file 1: The video describes the main features of Corona VRus Coaster and some examples of the functions that can be explored with the software (MP4 149 MB)

## Data Availability

The Corona VRus Coaster application, which enables immersive visualization of SARS-CoV-2 protein structures, is freely available for download at http://mduse.com/coronavruscoaster. The application includes preloaded access to 20 key SARS-CoV-2 protein structures, with the option to dynamically access an additional 2000 structures in real-time from the Protein Data Bank (PDB). Specific PDB IDs used in this study are listed in the manuscript. In addition to the VR version, a browser-based implementation has been developed using Unity WebGL, which allows non-VR access to the application via standard web browsers. This version is available at: https://mduse.com/coronavruscoaster/webgl/index.html.All structural data used in this work is publicly available and accessible through the PDB, ensuring reproducibility and data transparency.

## Declarations

### AI and AI-assisted technologies in the writing process

During the preparation of this work the authors used ChatGPT4o from OpenAI in order to improve language and readability. After using this tool/service, the authors reviewed and edited the content as needed and take full responsibility for the content of the publication.
